# Supplementary material for: Comprehensive Identification and Characterization of HML-9 Group in Chimpanzee Genome
Source: Viruses. 2024 May 31;16(6):892. doi: 10.3390/v16060892 (PMC11209481; doi:10.3390/v16060892)
Supplement: Supplementary file 1 [file viruses-16-00892-s001.zip › Supplementary Table S1.pdf]

Table S1 Conserved motif analysis of CERV-K HML-9 elements.

CERV-K HML-9 proviruses				
**Number**	**Logo**	**E-value**	**Sites**	**Width**
1	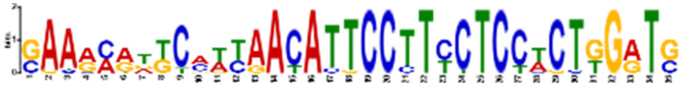	1.0e-186	19	35
2	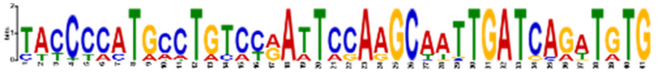	5.2e-245	25	41
3	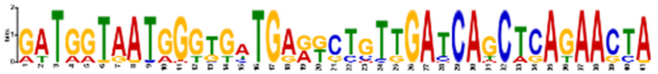	2.1e-232	25	41
4	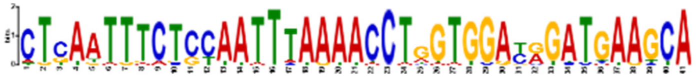	8.4e-218	22	41
5	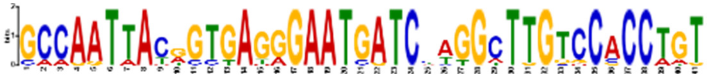	5.6e-229	23	41
6	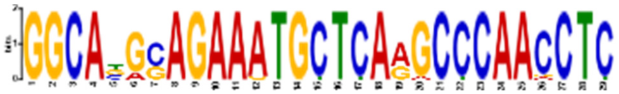	4.1e-154	20	29
7	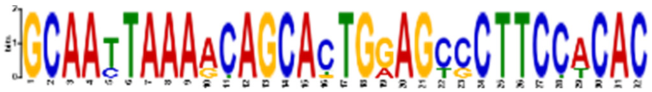	2.5e-151	17	32

8	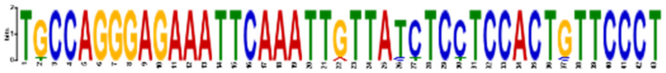	5.2e-145	13	43
9	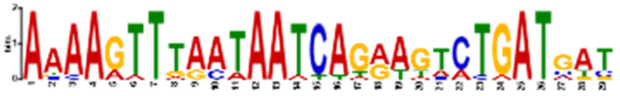	1.1e-133	14	29
10	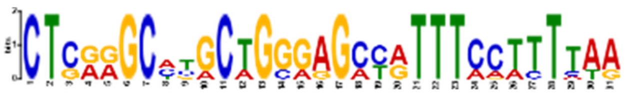	8.2e-140	22	31
**CERV-K HML-9 solo LTRs**				
**Number**	**Logo**	**E-value**	**Sites**	**Width**
1	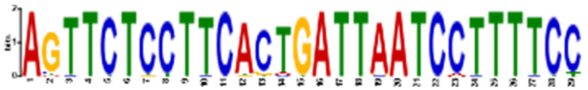	1.5e-418	38	29
2	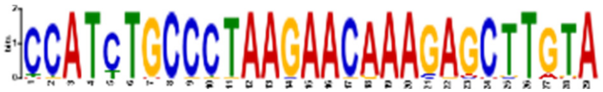	1.4e-415	38	29
3	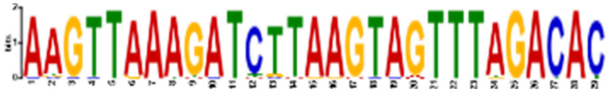	5.9e-414	38	29
4	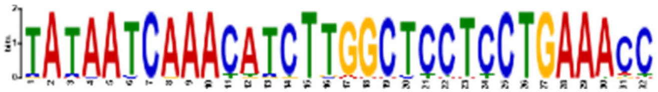	2.2e-454	38	32
5	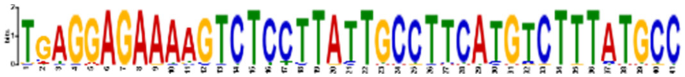	5.0e-600	38	41

6	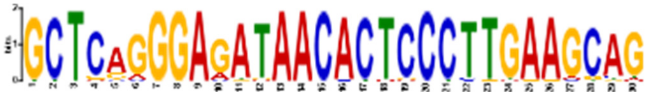	2.8e-389	37	30
7	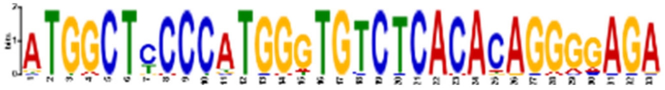	1.1e-383	33	33
8	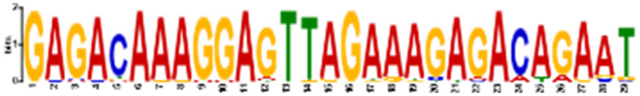	1.7e-369	37	29
9	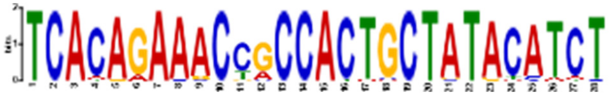	3.3e-366	37	28
10	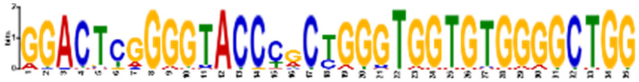	5.5e-429	36	35
**CERV-K HML-9 2-LTRs proviruses**				
**Number**	**Logo**	**E-value**	**Sites**	**Width**
1	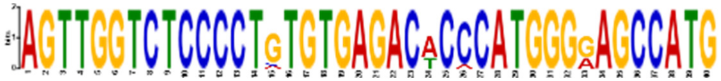	2.1e-160	12	40
2	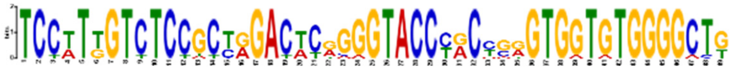	2.5e-152	12	49
3	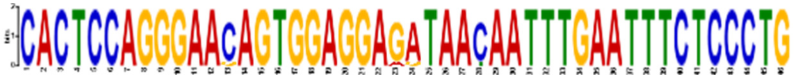	1.3e-144	10	46

4	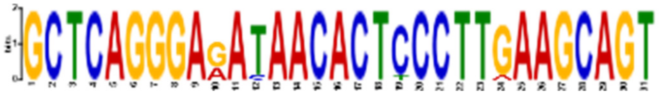	6.3e-111	12	31
5	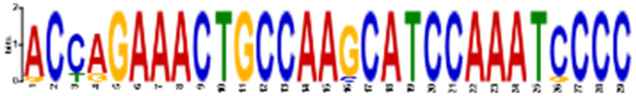	5.3e-100	12	29
6	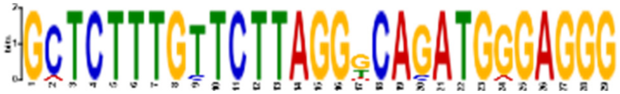	9.0e-099	12	29
7	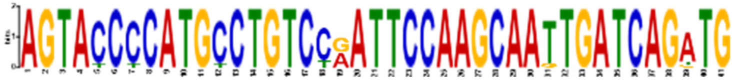	3.2e-155	12	41
8	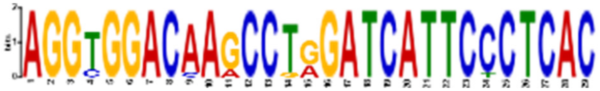	2.3e-097	12	29
9	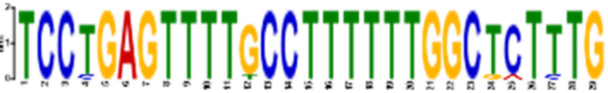	3.9e-097	12	29
10	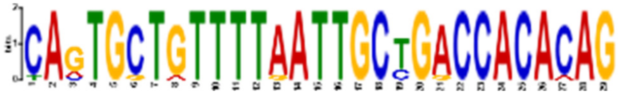	4.8e-090	12	29
number of motifs (10) found				
