# Supplementary figures and images for: Comprehensive Identification and Characterization of HML-9 Group in Chimpanzee Genome
Source: Viruses. 2024 May 31;16(6):892. doi: 10.3390/v16060892 (PMC11209481; doi:10.3390/v16060892)

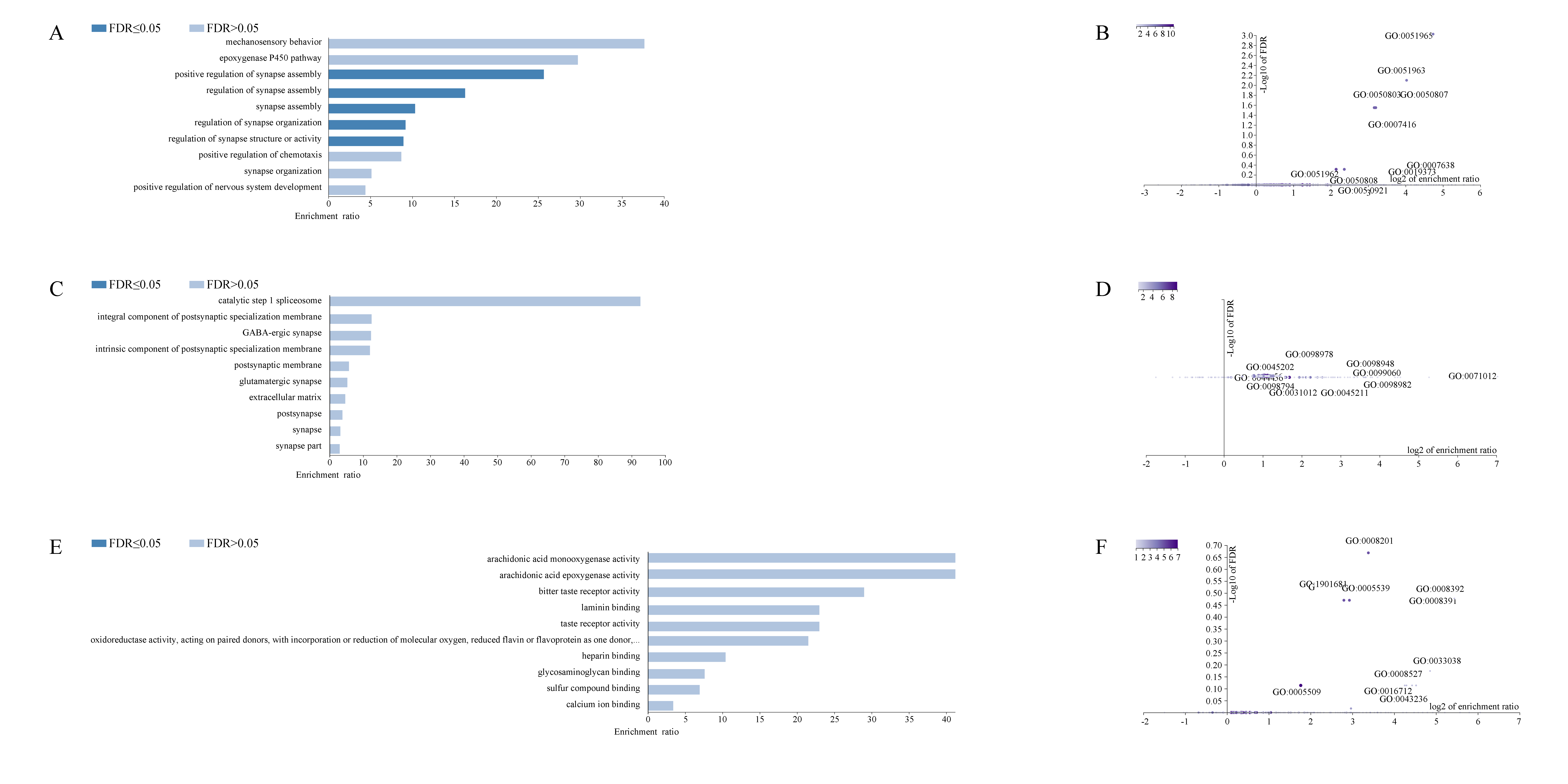

Supplement: Supplementary file 1 [file viruses-16-00892-s001.zip › Figure S5.tif]
